# Association of Retinoic Acid Receptors with Extracellular Matrix Accumulation in Rats with Renal Interstitial Fibrosis Disease

**DOI:** 10.3390/ijms131114073

**Published:** 2012-10-31

**Authors:** Yao-Bin Long, Yuan-Han Qin, Tian-Biao Zhou, Feng-Ying Lei

**Affiliations:** Department of Pediatrics, The First Affiliated Hospital of Guangxi Medical University, Nanning 530021, China; E-Mails: yaobinlong@yeah.net (Y.-B.L.); a126tianbiao@126.com (T.-B.Z.); leifengyinggx@yahoo.cn (F.-Y.L.)

**Keywords:** retinoic acid receptors, RARs, RARα, RARβ, RARγ, extracellular matrix, renal interstitial fibrosis

## Abstract

The nuclear retinoic acid receptors (RARs) function as ligand-dependent transcriptional regulators and include three subtypes (RARα, RARβ and RARγ), which control the expression of specific gene subsets subsequent to ligand binding and to strictly controlled phosphorylation processes. Extracellular matrix (ECM) accumulation is the most important characteristic of renal interstitial fibrosis (RIF). This study was performed to investigate whether RARs were associated with ECM accumulation in the progression of RIF in rats. Eighty Wistar male rats were divided into a sham operation group (SHO) and a model group subjected to unilateral ureteral obstruction (GU) at random; *n* = 40, respectively. The RIF disease in GU group was established by left ureteral ligation. The renal tissues were collected at two weeks and four weeks after surgery. Protein expressions of RARα, RARβ, RARγ, transforming growth factor-βl (TGF-β1), collagen-IV (Col-IV) and fibronectin (FN) were detected using immunohistochemical analysis, and mRNA expressions of RARα, RARβ, RARγ and TGF-β1 in renal tissue were detected by real time reverse transcription polymerase chain reaction. RIF index in renal interstitium was also calculated. When compared with those in SHO group, expressions of RARα and RARβ (protein and mRNA) were markedly reduced in the GU group (each *p* < 0.01). There was no marked difference for the expression of RARγ (protein and mRNA) between the SHO group and the GU group. The expressions of TGF-β1, Col-IV, FN and the RIF index in the GU group were markedly increased when compared with those in the SHO group (each *p* < 0.01). The protein expression of RARα/RARβ was negatively correlated with protein expression of TGF-β1, Col-IV or FN and the RIF index (all *p* < 0.01). In conclusion, the low expression of RARα/RARβ is associated with ECM accumulation in the progression of RIF in rats, suggesting that RARα/RARβ is a potentially therapeutic target for prevention of RIF.

## 1. Introduction

The retinoic acid receptors (RARs) function as ligand-dependent transcriptional regulators and include three subtypes (RARα, RARβ and RARγ), which control the expression of specific gene subsets subsequent to ligand binding and to strictly controlled phosphorylation processes [1,2]. RARα and RARβ appear at early stages of human embryonic development, and their expression is restricted to certain types of tissues [3]. In the past decades, there were lots of studies reporting that alterations of RARs expressions were associated with the development of some diseases, such as squamous-cell carcinoma [4], mammary carcinomas [5], esophageal cancer [6], *etc.* However, the role of RARs is complicated, and the mechanisms of RARs in the progression of diseases are not elucidated. Interestingly, Mendelsohn *et al.*[7] observed that the kidney development was impaired in compound mutants of RARs isotypes. RARs act as ligands for the metabolism of retinoic acid, and retinoic acid binds and activates the RARs and plays an essential role in many basic biological processes, such as cell proliferation, differentiation and apoptosis [8].

Renal interstitial fibrosis (RIF) is the final common pathway for chronic kidney disease, regardless of the etiology of the primary renal syndrome [9]. Moreover, interstitial fibrosis is the strongest morphologic predictor of clinical outcome and is most tightly linked to progression of disease [9,10]. Extensive accumulation of extracellular matrix (ECM) is the most important pathologic characteristic [11]. Collagen-IV (Col-IV) and fibronectin (FN) are the main components of ECM [12]. Furthermore, transforming growth factor-β1 (TGF-β1) is known to be one of the major mediators that leads to the accumulation of ECM [12]. Unilateral ureteral obstruction (UUO) is a well-characterized model of experimental obstructive nephropathy, culminating in ECM accumulation and interstitial fibrosis [13,14].

There were some studies reporting that alterations of RARs expression were associated with the development of renal diseases [15–18], and those studies were performed for the relationship between RARs and glomerulonephritis or podocytes injury. In our previous study [19], we found that all-trans retinoic acid could play a protective role against RIF in UUO rats. As the ligands for the all-trans retinoic acid, RARs might be associated with the risk of RIF. There is no any report studying the association of RARs with ECM accumulation, and there is no any report exploring the RARs expression in the renal tissue of UUO model. As those mentioned above, we drew a hypothesis that there was an association between RARs and ECM in RIF rats. This investigation was performed to explore whether RARs were associated with ECM accumulation in the progression of RIF in UUO rats.

## 2. Results

### 2.1. Renal Morphology

**Figure 1:**
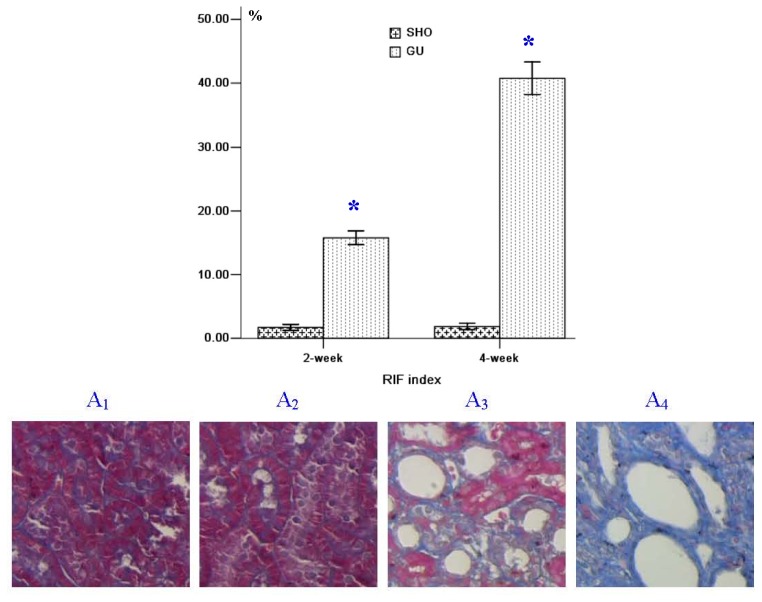
Tissue parameters in two groups. *p < 0.01 compared with SHO. The representative samples of Masson staining for the SHO group (A_1_: 2-week; A_2_: 4-week) and the GU group (A_3_: 2-week; A_4_: 4-week). SHO: sham operation group; GU: model group subjected to unilateral ureteral obstruction. Magnification: 400×.

More collagen deposition, fibroblast proliferation and diffused lymphoeytein filtration in the renal interstitium of the GU group were observed when compared with those in the SHO group (Figure 1). The index of RIF in the GU group was notably elevated when compared with that in the SHO group (*p* < 0.01; Figure 1). In the GU group, RIF index in week four was markedly increased compared with that in week 2 (4-week/2-week = 2.58).

### 2.2. Protein Expression of RARα, RARβ, RARγ, TGF-β1, Col-IV and FN in Renal Interstitium

**Figure 2:**
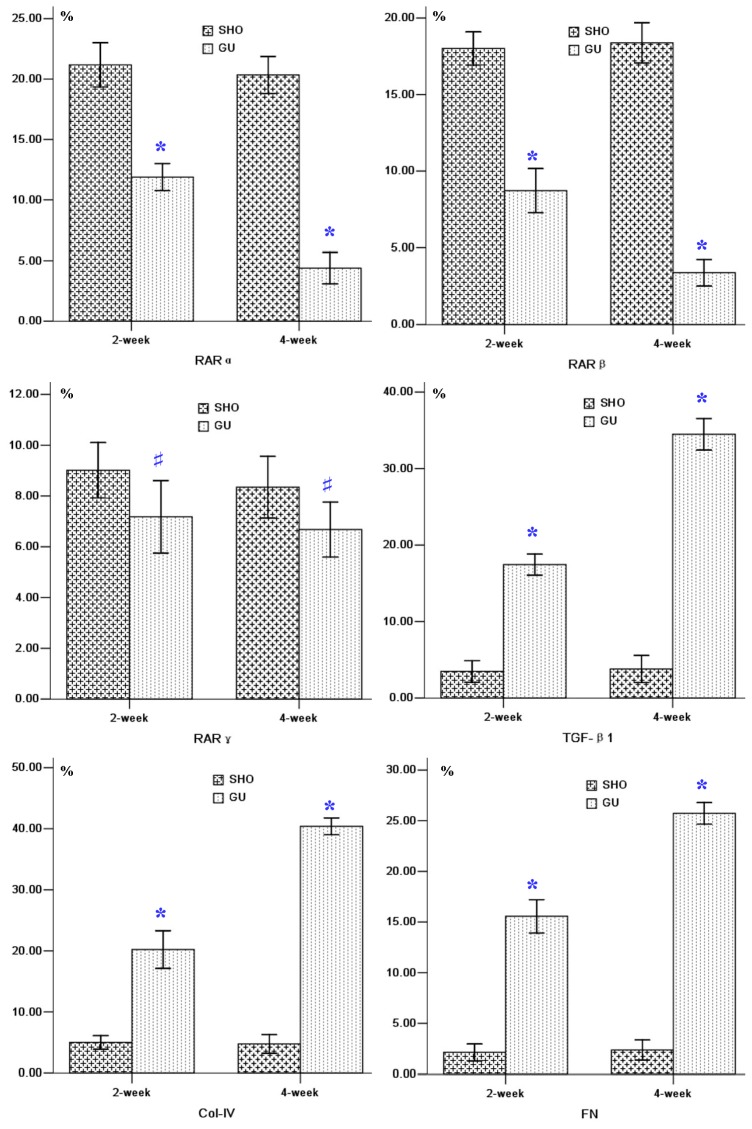

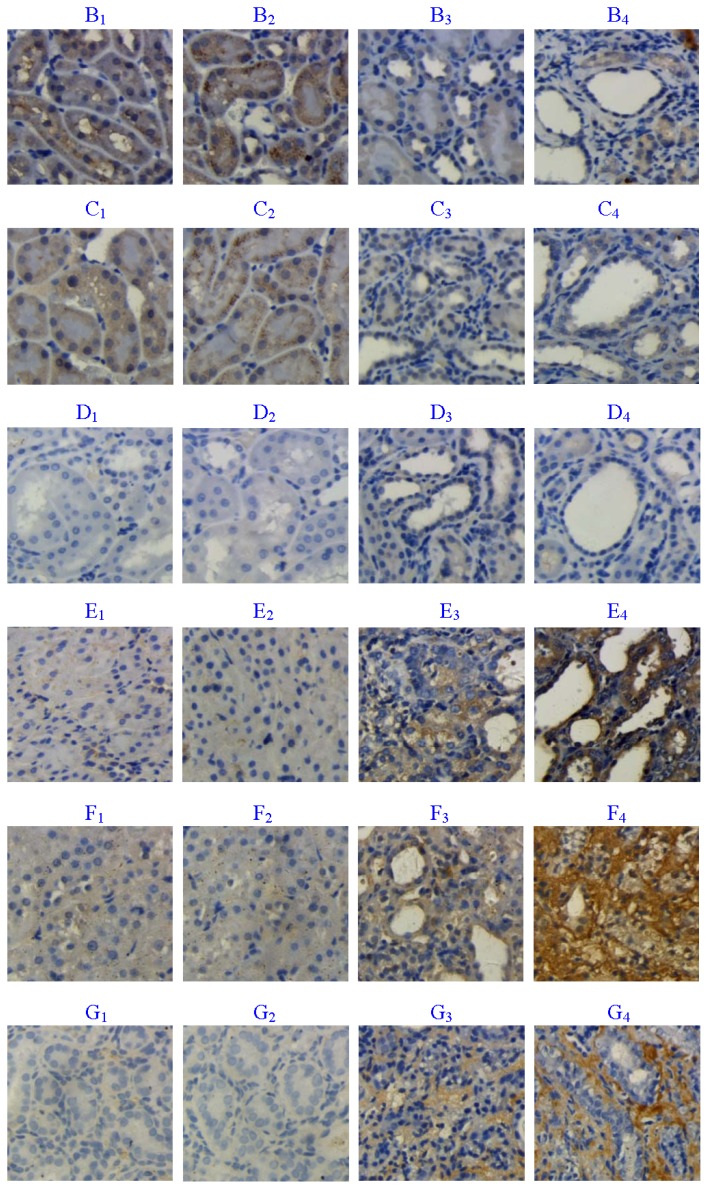
Tissue parameters in two groups. *p < 0.01 compared with SHO, # *p* > 0.05 compared with SHO. Representative samples of immunohistochemical staining for RARα (SHO: B_1_: 2-week, B_2_: 4-week; GU: B_3_: 2-week, B_4_: 4-week); RARβ (SHO: C_1_: 2-week, C_2_: 4-week; GU: C_3_: 2-week, C_4_: 4-week); RARγ (SHO: D_1_: 2-week, D_2_: 4-week; GU: D_3_: 2-week, D_4_: 4-week); TGF-β1 (SHO: E_1_: 2-week, E_2_: 4-week; GU: E_3_: 2-week, E_4_: 4-week); Col-IV (SHO: F_1_: 2-week, F_2_: 4-week; GU: F_3_: 2-week, F_4_: 4-week); and FN (SHO: G_1_: 2-week, G_2_: 4-week; GU: G_3_: 2-week, G_4_: 4-week) were observed in two groups. TGF-β1: transforming growth factor-βl; Col-IV: collagen-IV; FN: fibronectin; SHO: sham operation group; GU: model group subjected to unilateral ureteral obstruction. Magnification: 400×.

The RARα or RARβ staining in the GU group (B_3_ and B_4_ for RARα, and C_3_ and C_4_ for RARβ; Figure 2) was markedly lower when compared with that in the SHO group (B_1_ and B_2_ for RARα, and C_1_ and C_2_ for RARβ; Figure 2), especially in 4-week. The staining for RARγ in the GU group was similar with that in the SHO group. Positive stainings for TGF-βl, Col-IV and FN were stronger in the area of ECM in the GU group compared to those in the SHO group, especially in week four of the GU group. When compared with SHO, the protein expressions of RARα and RARβ were markedly reduced, and the protein expressions of TGF-βl, Col-IV and FN in renal interstitium were significantly increased in the GU group (all *p* < 0.01; Figure 2). There was no significant difference between the GU group and the SHO group for RARγ (*p* > 0.05; Figure 2). In the GU group, the expression of RARα or RARβ in week four was markedly weakened compared with that of week two (RARα: 4-week/2-week = 0.37; RARβ: 4-week/2-week = 0.39). The expressions of TGF-βl, Col-IV and FN in week four of GU group were markedly elevated when compared with week two (TGF-βl: 4-week/2-week = 1.98; Col-IV: 4-week/2-week = 2.00; FN: 4-week/2-week = 1.65).

### 2.3. mRNA Expressions of RARα, RARβ, RARγ and TGF-β1 in Renal Tissue

**Figure 3:**
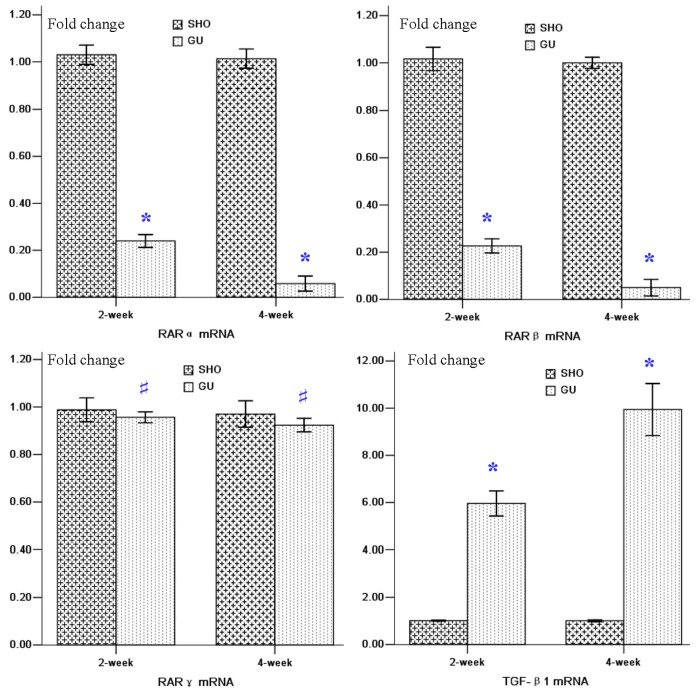
mRNA expressions of RARα, RARβ, RARγ and TGF-β1. *p < 0.01 compared with SHO, # *p* > 0.05 compared with SHO. SHO: sham operation group; GU: model group subjected to unilateral ureteral obstruction.

Renal tissue of the GU group showed consistently lower RARα and RARβ mRNA expressions when compared to those in SHO (*p* < 0.01; Figure 3). When compared with that in the SHO group, the mRNA expression of TGF-β1 in the GU group was markedly increased (*p* < 0.01; Figure 3). However, the difference for RARγ mRNA expression between the GU group and the SHO group was not statistically significant (*p* > 0.05; Figure 3). The mRNA expression of RARα or RARβ in week four of the GU group were remarkably reduced when compared with that in week two of the GU group (RARα: 4-week/2-week = 0.24; RARβ: 4-week/2-week = 0.22). The mRNA expression of TGF-βl in week four of GU group were remarkably elevated when compared with week two of GU group (4-week/2-week = 1.67).

### 2.4. Western-Blot Analysis for RARα, RARβ, RARγ and TGF-β1 in Renal Tissue

**Figure 4:**
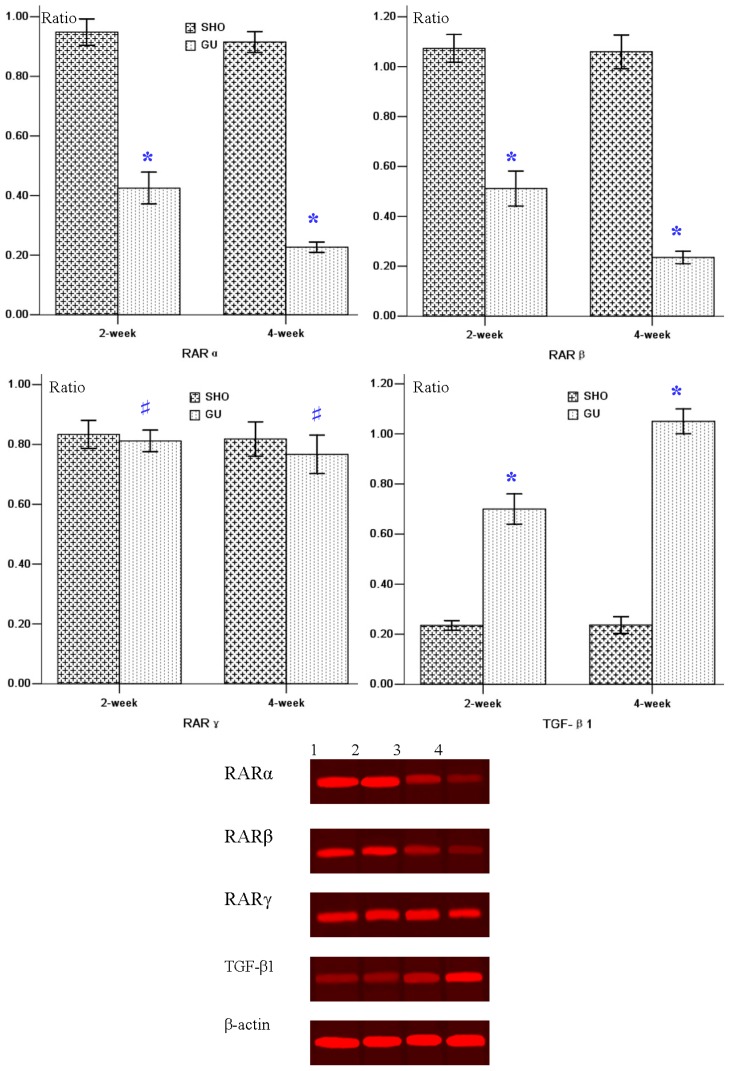
Statistical parameters of protein expressions of RARα, RARβ, RARγ and TGF-β1, and the western-blot figures in two groups. 1: 2-week of SHO; 2: 4-week of SHO; 3: 2-week of GU; 4: 4-week of GU. *p < 0.01 compared with SHO, # *p* > 0.05 compared with SHO. SHO: sham operation group; GU: model group subjected to unilateral ureteral obstruction.

Lower RARα/RARβ protein expression in GU group was observed when compared to that in the SHO group (*p* < 0.01; Figure 4). However, the protein expression of TGF-β1 in UUO rats was much higher compared with that in the SHO group (*p* < 0.01; Figure 4). The difference of RARγ expression between the GU group and the SHO group was not statistically significant (*p* > 0.05; Figure 4). The protein expression of RARα or RARβ in week four of the GU group was markedly reduced when compared with that in week two of the GU group (RARα: 4-week/2-week = 0.53; RARβ: 4-week/2-week = 0.46). The protein expression of TGF-βl in week four of the GU group was notably elevated when compared with week two of the GU group (4-week/2-week = 1.50).

### 2.5. Correlation Analysis

RARα protein level was negatively correlated with RIF index, TGF-βl, Col-IV or FN (*r* = −0.833, −0.763, −0.825 and −0.918, respectively; each *p* < 0.01). RARβ expression was negatively correlated with RIF index, TGF-βl, Col-IV or FN (*r* = −0.728, −0.736, −0.818 and −0.784, respectively; each *p* < 0.01). There was no correlation between RARγ and RIF index, TGF-βl, Col-IV or FN (*r* = 0.145, 0.261, 0.126 and 0.225, respectively; each *p* > 0.01).

## 3. Discussion

The ECM provides structural support by serving as a scaffold for cells, and as such, the ECM maintains normal tissue homeostasis and mediates the repair response following injury [20]. However, the accumulation of ECM is the most important pathologic characteristic of RIF, and the mechanism for the ECM accumulation is not elucidated at present. Col-IV and FN are the main components of the ECM [12]. Furthermore, TGF-βl is a risk factor to induce the ECM accumulation. In this study, we found that the expressions of TGF-βl, Col-IV and FN in the renal interstitium of the GU group were increased compared to those in the renal interstitium of the SHO group. The results in this study were similar with those in our previous report [21]. It indicated that the RIF model in our study was established successfully.

The alterations of RARs expressions were associated with the development of some diseases. However, in different tissues/cells, the RARs expressions were different. The association of RARs with the development of some diseases is conflicting in the past decades. We speculated that the roles of RARs in the different tissues/cells were different. In our previous studies [19,22], we found that all-trans retinoic acid could play a protective role against RIF in UUO rats. We speculated that RARs, the ligands for the all-trans retinoic acid, might be associated with the risk of RIF. In this investigation, we first reported the expressions of RARs in the renal tissue of RIF rats induced by UUO.

Lower expression of RARα was found in the RIF rats when compared with that in the normal control rats. We also found that RARα protein level was negatively correlated with RIF index, TGF-βl, Col-IV or FN. It indicated that the low expression of RARα was associated with the progression of RIF in UUO rats. There were some reports exploring the association of RARα expression with renal diseases. Schaier *et al.*[15] conducted a study in acute anti-Thy1.1 glomerulonephritis (Thy-GN) of the rat, and found that the expression of RARα was markedly increased in vehicle-treated nephritic glomeruli compared to nonnephritic controls. He *et al.*[16] found that HIV infection significantly suppressed expression of RARαin podocytes and induced podocyte proliferation. Ratnam *et al.*[17] suggested that knockout of RARα caused more severe podocyte injury and kidney damage in HIV-1 transgenic mice (Tg26), and RARα likely acted as an endogenous protective pathway to prevent the development and the progression of kidney disease. In different renal disease models, the association of RARα expression with renal diseases was different. Those studies mentioned above were performed on glomeruli diseases, and there was no any study exploring the role of RARα in the pathogenesis of RIF. More studies for the relationship between RARα and should be performed in the future.

Less expression of RARβ in the RIF rats was found when compared with that in control rats. RARβ expression was negatively correlated with RIF index, TGF-βl, Col-IV or FN. It indicated that less expression of RARα was associated with the progression of RIF in UUO rats. There were some reports studying the association of RARβ expression with renal diseases. Zhong *et al.*[18] found that the RARβ in the glomeruli of Tg26-vehicle mice was reduced compared to that in the wild type mice. He *et al.*[16] found that HIV infection significantly suppressed expression of RARβ in podocytes and induced podocyte proliferation. However, Schaier *et al.*[15] conducted a study in acute Thy-GN of the rat and found that the expression of RARβ was markedly increased in vehicle-treated nephritic glomeruli compared to nonnephritic controls. The association of RARα expression with renal diseases was also conflicting in different renal diseases. Furthermore, those studies mentioned above were performed on glomeruli diseases, and there was not any study exploring the role of RARβ in the pathogenesis of RIF. Further studies for the relationship between RARβ and the renal diseases should be conducted in the future.

The association of RARγ expression with RIF development was not found in this study, and there was no correlation between RARγ and RIF index, TGF-βl, Col-IV, or FN. It showed that RARγ expression was not associated with the RIF development in UUO rats. There was only one report studying the role of RARγ on the development of renal diseases. Schaier *et al.*[15] conducted a study in acute Thy-GN of the rat, and found that the expression of RARγ was lower in nephritic glomeruli of vehicle-treated rats compared to those of nonnephritic controls. However, Schaier *et al.*[15] conducted the study in glomerulus disease rats and our study was conducted in RIF rats. In the different renal diseases models, the expression of RARγ might be different. Those reports mentioned above were conducted in glomeruli diseases, and there was no any report exploring the role of RARγ in the pathogenesis of RIF. Further studies for the relationship between RARγ and the renal diseases should be conducted in the future.

There was rare report in the past studying the association of RARs with the ECM accumulation. In this study, we first report that RARα or RARβ was associated with the accumulation of ECM in RIF rats induced by UUO. However, further study should be conducted in the future.

## 4. Materials and Methods

### 4.1. Animal Model

The Animal Care and Use Committee of Guangxi Medical University approved all protocols in this experiment. Eighty Wistar male rats (6-week-old) were purchased from the Experimental Animal center of Guangxi Medical University, Nanning, China. Those 80 rats were divided into two groups at random: a sham operation group (SHO) and a model group subjected to unilateral ureteral obstruction (GU). The ureter was ligated at approximately 1 cm below the renal hilum with 3-0 silk suture. The abdominal wound was closed, and rats returned to the cages. Control rats underwent abdominal incision and approximation with no ligation of the ureter [23,24]. Twenty rats in the two groups were killed on week two and week four after surgery, respectively, and their renal tissue was collected for histological and molecular biology determination.

### 4.2. Renal Morphology

The renal tissues were dehydrated through a graded ethanol series after 10% neutral formaldehyde fixation and embedded in paraffin. Sections were prepared on a microtome and stained with Masson’s trichrome staining. Renal pathology was observed by light microscope, and the severity of the renal lesion was presented by the RIF index. Blue granular or linear deposits were interpreted as positive areas for collagens staining. Semi-quantitative evaluation was performed by computer-assisted image analysis (DMR+Q550, Leica Co., Germany). The area of positive staining of fibrosis was measured at 400-folds original magnification in twenty fields (ignoring the fields containing glomerular parts), which were selected from coded sections for each rat at random [25].

### 4.3. Immunohistochemical Analysis of the Protein Expressions of RARα, RARβ, RARγ, TGF-β1, Col-IV and FN

Renal tissue fixed with 4% buffered paraformaldehyde was embedded in paraffin, and 4 μm-thick sections were stained. The positive area was measured quantitatively using a computer-aided manipulator (DMR+Q550, Leica Co., Solms, Germany). For immunohistochemical analysis of RARα, RARβ, RARγ, TGF-β1, Col- IV and FN, the sections were deparaffinized, washed with PBS and treated with 3% H_2_O_2_ in methanol for 10 min. All sections were then incubated with anti-RARα antibody (Abcam, Co., Cambridge, MA, USA), anti-RARβ antibody (Abcam, Co., Cambridge, MA, USA), anti-RARγ antibody (Abcam, Co., Cambridge, MA, USA), anti-TGF-β1 antibody (Abcam, Co., Cambridge, MA, USA), anti-Col-IV antibody (Abcam, Co., Cambridge, MA, USA) and anti-FN antibody (Abcam, Co., Cambridge, MA, USA), respectively. After incubation with second antibody immunoglobulin, the sections were stained with diaminobenizidine. The positive area of RARα, RARβ, RARγ, TGF-β1, Col-IV or FN in renal tissue was measured. During evaluation of the interstitial areas, fields containing glomerular parts were ignored. All the evaluations were performed by two of the authors blinded to the experimental code.

### 4.4. Real Time Reverse Transcription Polymerase Chain Reaction to Detect mRNA Expressions of RARα, RARβ, RARγ and TGF-β1 in Renal Tissue

Renal tissue was homogenized and total RNA was extracted with TRIzol (Beijing Tiangen, Co., Beijing, China). Ultraviolet spectrophotometer measured the absorbance, and agarose gel electrophoresis confirmed that there had been no degradation of RNA by visualizing the 18S and 28S RNA bands under ultraviolet light [12,26]. Primers were designed according to primer design principles by Primer Premier 5.0. One microgram total RNA from the renal tissue of each rat was reverse transcribed into cDNA with an ExScript RT reagent kit (Fermentas, MBI). RARα, RARβ, RARγ, TGF-β1 and β-actin were amplified with SYBR Premix Ex Taq (Roche Inc., Basel, Switzerland). Gene expression of β-actin was also measured in each sample and used as an internal control for loading and reverse transcription efficiency. The analysis for each sample was performed in triplicate. The average threshold cycle (Ct, the cycles of template amplification to the threshold) was worked out as the value of each sample. The data for fold change was analyzed using 2^−ΔΔCt^[26,27].

### 4.5. Western-Blot Analysis for RARα, RARβ, RARγ, and TGF-β1 in Renal Tissue

The renal tissue homogenized and the total protein was isolated with radio immunoprecipitation assay lysis buffer and a mixture of protease inhibitors (PMSF, Sigma Chemical Co., St. Louis, MO, USA). After protein concentration quantitation with the modified Bradford assay (Bio-Rad Laboratories, Hercules, CA, USA) [28], 40 mg total protein was then used for Western blotting with primary antibodies against RARα, RARβ, RARγ and TGF-β1. Membranes were imaged by the LiCor Odyssey scanner. Boxes were manually placed around each band of interest, which returned near-infrared fluorescent values of raw intensity with intra-lane background subtracted using Odyssey 3.0 analytical software (Li-Cor, Lincoln, NE, USA) [29]. β-actin was used as an internal control, and it was detected by β-actin antibody (Abcam).

### 4.6. Statistical Analysis

The data are shown as mean ± standard deviation. Independent-Samples *t*-Test was performed to determine the differences between the SHO group and the GU group, and the Pearson’s correlation coefficients were used to determine the relationships between the indicators for detection. A value of *p* < 0.05 was considered as significant. Statistical analysis was performed using the statistical package for social studies SPSS version 13.0 (SPSS, Chicago, IL, USA).

## 5. Conclusion

Less expression of RARα or RARβ is associated with the ECM accumulation in the progression of RIF in UUO rats, although the detailed mechanisms are not fully understood. So, how to up-regulate the RARα or RARβ expression might be very important for prevention of RIF, and RARα or RARβ might be a potential therapeutic target for prevention of the ECM accumulation in the progression of RIF. This observation might offer some new insights to prevent the progression of RIF. However, cells culture, such as RTEC, and inhibition of the signaling pathway for RARα or RARβ need to be conducted to explore its detailed mechanism.
